# Does the use of shortened stems reduce early femoral complications in total hip arthroplasty using the direct anterior approach?

**DOI:** 10.1186/s42836-025-00317-y

**Published:** 2025-07-01

**Authors:** François Fauré, Cécile Batailler, Constant Foissey, Elvire Servien, Sébastien Lustig

**Affiliations:** 1 Orthopaedics Surgery and Sports Medicine Department, FIFA Medical Center of Excellence, Croix-Rousse Hospital, Lyon University Hospital, 69004 Lyon, France; 2 IFSTTAR, LBMC, UMR_T9406, Univ Lyon, Claude Bernard Lyon 1 University, 69622, 69008 Lyon, France; 3 LIBM–EA 7424, Interuniversity Laboratory of Biology of Mobility, Claude Bernard Lyon 1 University, 69100 Villeurbanne, France

**Keywords:** Total hip arthroplasty, Direct anterior approach, Great trochanter fracture, Shortened stem, Complications

## Abstract

**Introduction:**

The choice of femoral stem design during total hip arthroplasty (THA) through the Direct Anterior Approach (DAA) is critical. Shortened stems offer potential benefits such as bone preservation and reduced stress shielding. This study aimed to compare early complications at one year of follow-up between shortened and standard stems in DAA THA.

**Methods:**

A retrospective monocentric case–control study included patients undergoing DAA THA from 2013 to 2023. Two cohorts were analyzed: 537 THA with standard stems and 346 THA with shortened stems. Three hundred forty-three patients in each group were matched (1:1) based on age, sex, and Body Mass Index (BMI). Two independent observers assessed femoral complications at one year. Femoral stem positioning was measured.

**Results:**

The mean follow-up was 12 ± 0.5 months. The mean age was 64.1 ± 11.7 years. The mean BMI was 26.4 ± 4.4 kg/m^2^. Shortened stems showed a significantly lower rate of femoral complications (1.4% vs. 5.5%, *P* = 0.005), particularly for the GT fractures (*P* = 0.006). In the shortened group, stem alignment was neutral in 69% of cases, varus in 27%, and valgus in 4%.

**Conclusion:**

Shortened stems in DAA THA were associated with a lower rate of femoral complications, particularly fewer GT fractures. Although shortened stems were more often positioned in varus, this did not impact short-term complication rates.

**Trial Registration:**

The Advisory Committee on Research Information Processing in the Field of Health (CCTIRS) approved this study on June 4, 2015 (Study ID 15–430).

Video Abstract

**Supplementary Information:**

The online version contains supplementary material available at 10.1186/s42836-025-00317-y.

## Introduction

Total hip arthroplasty (THA) using the Direct Anterior Approach (DAA) has gained popularity due to the potential for quicker recovery, reduced soft tissue damage, and lower risk of dislocation [1]. One of the difficulties of this approach is the femoral exposure. For this reason, some teams use traction tables to facilitate femoral preparation. However, this technique does not allow control of leg length discrepancy and increases the risk of intraoperative fracture [2]. The technical mastery of this approach requires a learning curve [3–5] and may cause some complications, such as fractures of the greater trochanter (GT) or femoral diaphysis [6–9]. A preoperative unfavorable ambulatory condition, a diagnosis of rheumatoid arthritis, a high DORR ratio, and a low Femoral Neck Cut Ratio FNCR are associated with an increased risk of fracture by Hartford [8]. Foissey and Greco found that an age of > 70 years and female gender are risk factors for fractures in THA with DAA [3, 10].

One critical aspect of THA outcomes is the choice of femoral stem design. Shortened stems have emerged as an alternative to standard stems, promising advantages such as preserving bone stock and facilitating minimally invasive techniques, reducing the risk of proximal stress shielding and thigh pain, and decreasing bone loss around the lesser trochanter [11–15]. The shortened stems also facilitate femoral exposure and preparation. Some shortened stem designs have already been evaluated, but each design is different and requires further assessment. The emergence of stems with a collar has demonstrated safety in stem subsidence and reduction in peri-prosthetic fractures [16]. Combining a shortened stem and a collar could be very interesting during DAA THA.

The aim of the study was to assess early complications and revisions at one year of follow-up with shortened stems versus standard stems by DAA. The hypothesis was that the shortened stems decreased the risk of early femoral complications compared to standard stems by DAA.

## Materials and methods

### Patients

This retrospective single-center case–control study included all patients who underwent THA through DAA from May 2013 to March 2023. The inclusion criteria were primary DAA THA and the same uncemented collared stem in their standard or shortened versions. Exclusion criteria were body mass index (BMI) > 45 kg/m^2^, abnormal hip anatomy requiring complex THA (e.g., congenital hip dysplasia), elderly patients (> 90 years old), Dorr C-classified femurs, associated bone procedures, and minor patients age < 18 years old. The minimum follow-up required was 1 year postoperative. Two cohorts were assessed: a first consecutive cohort of 537 THA using the same uncemented standard stem with collar (Targos stem (Lepine, Genay, France)) and a second cohort of 346 THA using the same uncemented shortened stem with collar (Targos Mini stem (Lepine, Genay, France)). The Targos Mini was introduced in our department in 2018. A single senior surgeon performed all surgeries. The surgeon had already accumulated over 10 years of experience with the DAA before the start of the study.

**Fig. 1:**
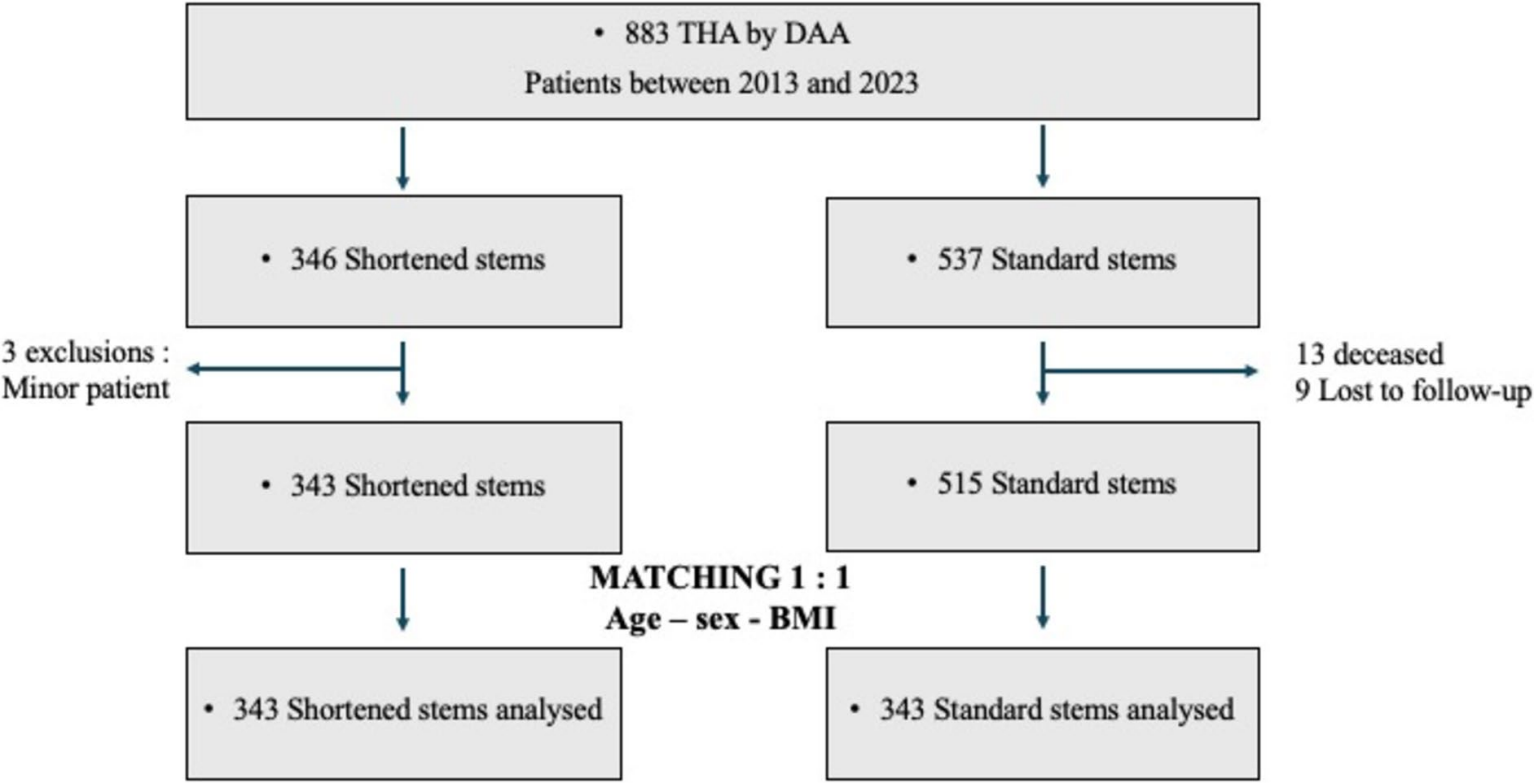
Flowchart

Three minor patients were excluded from the 346 shortened stems. Thus, 343 shortened femoral stems from 318 patients (28 bilateral THAs) were included in this study. During the same period, 537 THA procedures using standard stems were performed. Four patients didn’t have preoperative X-rays, 13 deceased within 2 months postoperatively, and five were lost to follow-up. From the cohort, 343 patients were included in each group and matched (1:1) with age, sex, and BMI. The mean follow-up was 12 ± 0.5 months. (Fig. 1 and Table 1).

**Table 1:** Patient and demographic data

Parameters	Shortened stem (*n*: 343)	Standard stem (*n*: 343)	*P*-value
Gender (%M)	147 (46%)	151 (44%)	0.99
Mean age (years)	64.1 ± 12 [18–87]	65.0 ± 12 [18–88]	0.99
Mean BMI (kg/m^2^)	26.2 ± 4.7 [15.6–40.5]	26.0 ± 4.0 [17.0–41.0]	0.75
Etiology			
Primitive	289 (84%)	268 (78%)	0.53
ONFH	38 (11%)	41 (12%)	0.81
Dysplasia	16 (5%)	21 (6%)	0.5
Other	0	13 (4%)	0.06

ONFH: Osteonecrosis of femoral head

Other: Post-traumatic (*n* = 5), osteochondritis (*n* = 2), acetabular fracture (*n* = 2), epiphysiolysis (*n* = 2), Post osteotomy (*n* = 1), Osteomyelitis (*n* = 1)

### Surgical technique

The same standardized Hueter Gaine approach was used for all patients. The DAA was performed supine without a traction table, as described by Lustig [17]. The fluoroscopic examination was systematically performed at the end of the surgery.

### Implant design

**Fig. 2:**
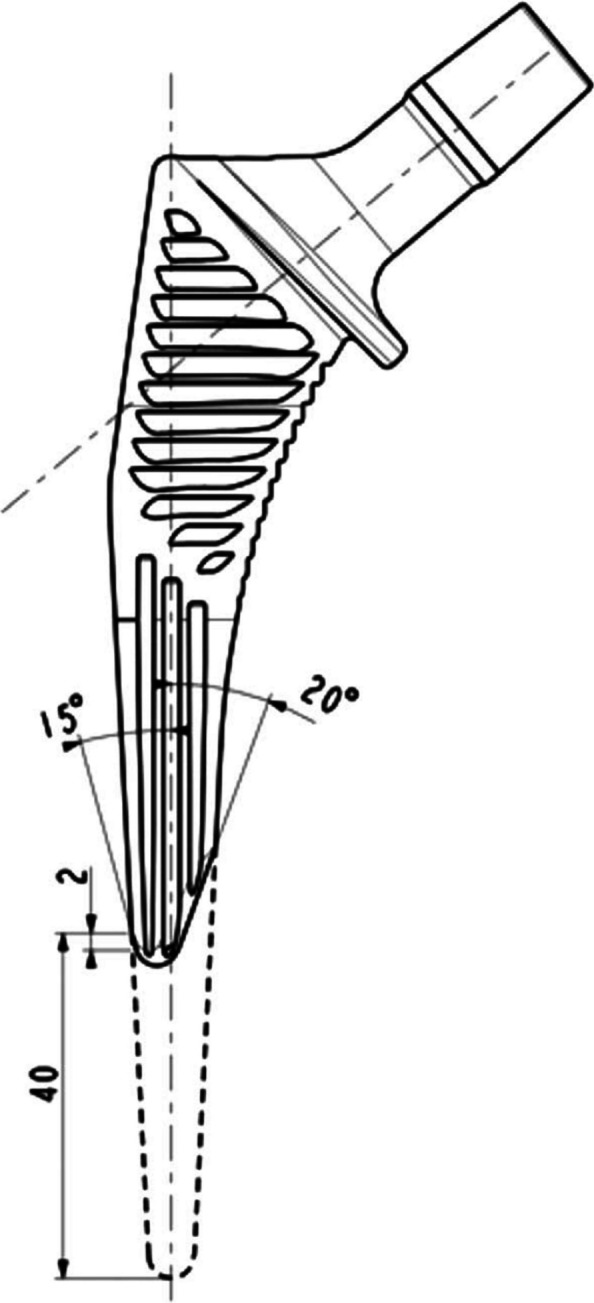
Design of the Targos Mini stem compared to the standard stem

Two femoral stems were analyzed: an uncemented standard stem with collar (Targos (Lepine®, Genay, France)) and an uncemented shortened stem with collar (Targos Mini (Lepine®, Genay, France)). The design includes a proximal trapezoidal cross-section intended to resist axial stresses and promote initial stability, and a tapered distal stem that is quadrangular provides a decreasing stiffness gradient to reduce the elastic mismatch between the prosthesis and bone. Each implant has a nonporous, fully hydroxyapatite coating on a forged titanium alloy stem. The standard and the shortened stems feature a triple taper geometry, mediolateral, anteroposterior, and distal tapering designed to optimize metaphyseal-diaphyseal press-fit fixation while minimizing stress shielding. The only difference between the two stem designs was their respective lengths. The shortened stem was 40 mm shorter than the standard stem. The metaphyseal part was the same between these implants (Fig. 2).

### Clinical and radiological evaluation

After surgery, patients were systematically reviewed for radio-clinical follow-up at two months and one year. The radiographs included an anteroposterior and lateral view of the hip.

**Fig. 3:**
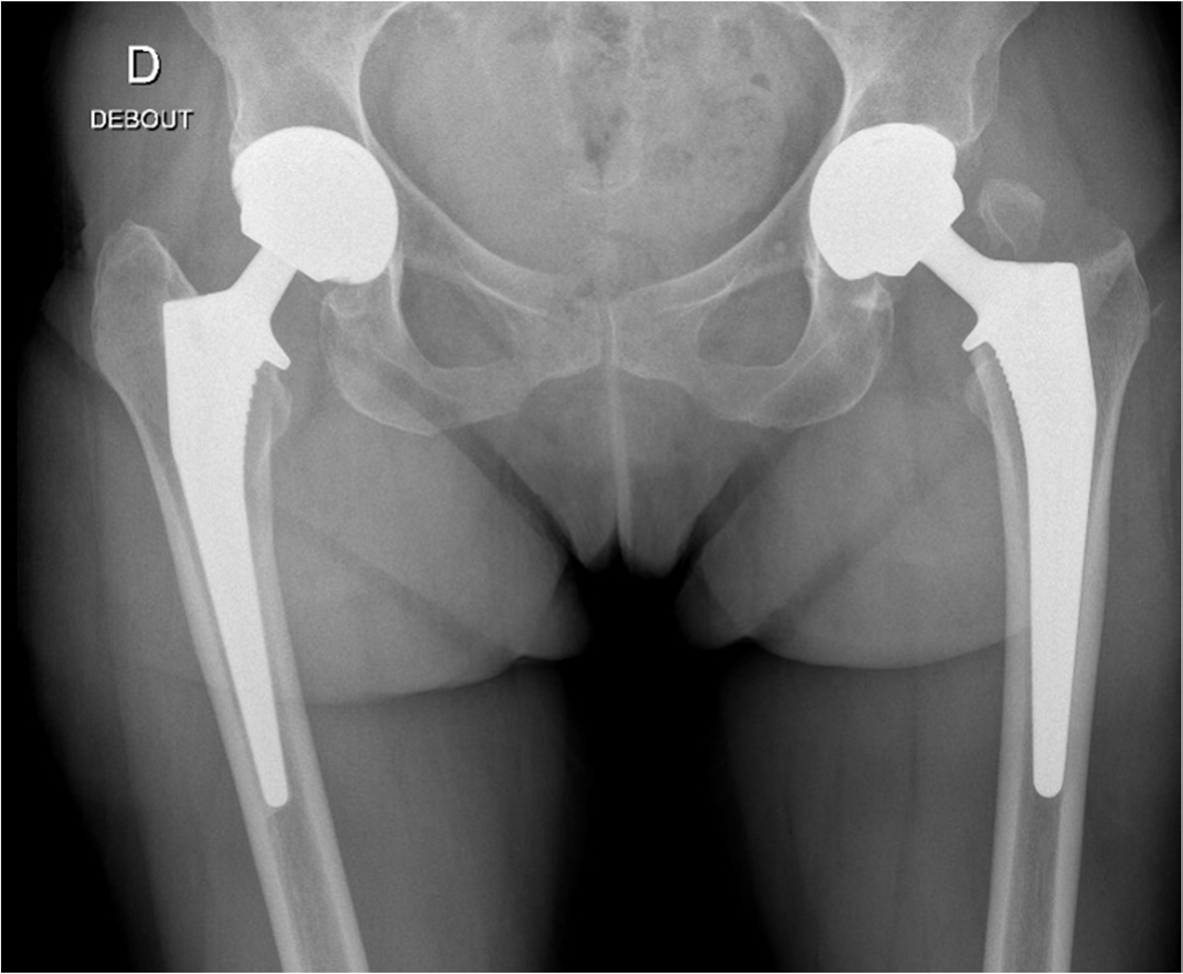
Great Trochanter Fracture with a standard stem

Two observers recorded postoperative radiological femoral complications (GT fractures, periprosthetic femoral fractures, loosening, secondary displacement) (Fig. 3).

**Fig. 4:**
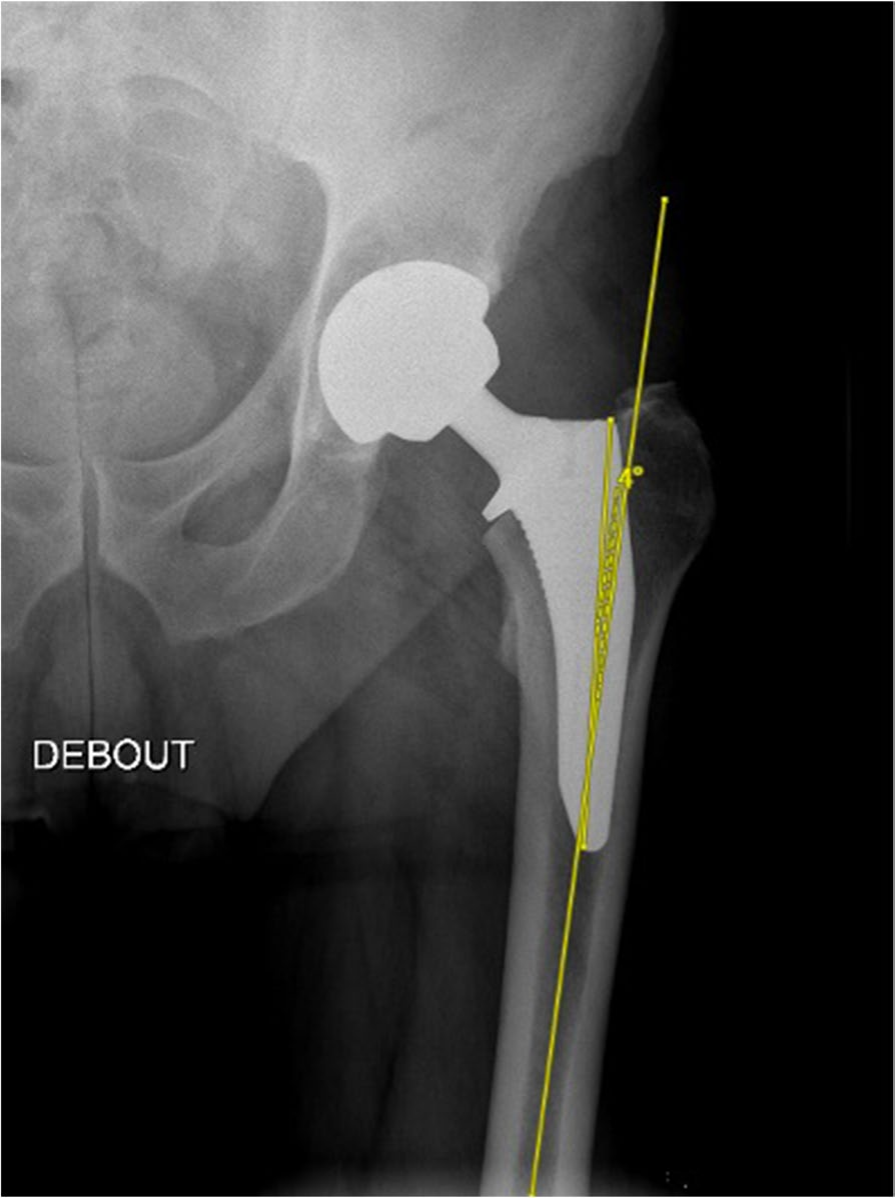
Measurement of the alignment of the femoral stem

The same observer measured femoral stem coronal alignment on an anteroposterior pelvic view one year postoperatively. Inter-observer reliability was assessed for 10 patients in each group. Positioning of the stem was determined by measuring the angle formed between the prosthesis’s long axis and the femur's long axis. Positioning was recorded as positive for varus and negative for valgus. The threshold for malalignment was considered at +3° for varus alignment [18] (Fig. 4)*.*

### Statistical analysis

The statistical analysis was performed with the online software EasyMedStat® (https://www.easymedstat.com; Neuilly-sur-Seine, France). The continuous variables were averaged and reported with means, standard deviations, and ranges. They were compared using the Student *t*-test or the Wilcoxon nonparametric test. The categorical variables were compared using a Fisher’s exact test. A *P*-value < 0.05 was considered statistically significant for all analyses.

*Ethical approval.*

All procedures were performed in accordance with the ethical standards of the institutional and/or national research committee, the 1964 Helsinki declaration and its later amendments, or comparable ethical standards.

For this study, formal consent was not required. The Advisory Committee on Research Information Processing in the Field of Health (CCTIRS) approved this study on June 4, 2015 (Study ID 15–430). All data were fully anonymized before their access. The ethics committee did not require informed consent.

## Results

### Femoral complications

Five femoral complications (1.4%) were recorded in the shortened group: 2 GT fractures, one diaphyseal fracture, one stem subsidence, and one psoas tenotomy. One fracture was seen at the time of the surgery (GT fracture), one at the immediate postoperative radiographs (GT fracture), one within the first 2 months after surgery (diaphyseal fracture confirmed on CT scan), and one at 6 months after surgery (subsidence). Three occurred in women at an average age of 71 (65–83). For these patients, all implants were in the neutral position. Among these complications, only one patient underwent surgical revision with a unipolar change of the femoral stem; the other complications were treated orthopedically with non-weight bearing and had no consequences on postoperative follow-up.

Nineteen femoral complications (5.5%) were observed in the control group: 13 GT fractures (4.3%), four femoral fractures (1.1%), and two calcar fractures (0.5%) (Table 2). The complication rate was significantly higher in the control group (*P* = 0.005), particularly for the GT fractures (*P* = 0.006).

**Table 2:** Complications

	**Shortened stems**	**Standard stems**	***P*-value**	**Implant Position Shortened/Std (degrees)**
**Femoral complication**	**5**	**19**	**0.005**	
Psoas tenotomy	1	0	0.99	
GT fracture	2	13	0.006	Shortened: 1.6° [1.4–1.7]; STD: 1.85° [0–5]
Femoral fracture	1	4	0.37	
Stable calcar fracture	0	2	0.50	
Subsidence	1	0	0.99	
**Acetabular complication**				
Acetabular cup displacement	2	3	0.67	
Dislocation	1	0	0.99	
Ceramic fracture	2	0	0.50	

### Alignment of the femoral stem

The average alignment of the shortened stem was 2.0° ± 1.9 [−4.3;8.6]. Most shortened stems were neutral (69%), 27% were varus, and 4% were valgus. The range varied from −4.3° to 8.6°. For standard stems, the mean alignment was 1.9° ± 1.5 [−2;9]. In the control group, 80% were neutral, 17% were varus, and 3% were valgus. There were statistically more implants positioned in varus in the shortened group compared to the control group (*P* = 0.007). The difference in mean alignment between the two groups was minimal (2.0° vs. 1.9°, *P* = 0.012). (Table 3).

**Table 3:** Implant Position

	**Shortened Stem (*n* = 343)**	**Standard Stem (*n* = 343)**	***P*-value**
Neutral	236, 69%	273, 80%	0.24
Varus	93, 27%	59, 17%	0.007
Valgus	14, 4%	11, 3%	0.55
Average	2.0° ± 1.9 [−4.3–8.6]	1.9° ± 1.5 [−2–9]	0.012

All GT fractures observed in the shortened group had a femoral implant in a neutral position (1.4° and 1.7°). In the standard group, only one fracture was associated with an implant in varus alignment (average: 1.8° [0–5]).

## Discussion

The main finding of this study was the low rate of femoral complications associated with THA using shortened stems by DAA, particularly GT fractures. Shortened stems offered advantages over standard stems in reducing femoral complications.

In our study, the overall complication rate was 1.4% for THA using shortened stems. Specifically, GT fractures were significantly lower (0.57%) compared to those using standard stems (2.4%) [3].

These findings align with previous studies, short-term complications range between 0 and 2.4% [8, 19–22]. Hartford et al. [8] found a 2.2% GT fracture rate in their study, which included both standard stems and shortened stems of different designs performed by DAA. Dietrich et al. [23] also observed a significant decrease in the peroperative and short-term complication rates with the use of short stems: Fitmore stem (Zimmer), AMIStem (Medacta), compared to standard stems: Quadra-H stem (Medacta) (11.8% vs. 4.4%), performed by a modified Hueter approach. However, Tamaki’s study [24] reported conflicting results, with higher complication rates with short stems (1.2% vs. 0.8%). the stems studied were from the same manufacturer (Biomet, Warsaw, IN), featuring a cementless tapered-wedge design with either a short or standard length: Taperloc Microplasty (short stem) and Taperloc (standard stem). In the literature, it is crucial to distinguish the kind of short or shortened femoral stem. Their fixation and design are very variable, according to the manufacturer. In a retrospective study by Katakam et al. [25], the authors compared three designs of the Taperloc® Complete Hip System (full-length, reduced distal profile, and short-length) in total hip arthroplasty performed via posterior approach. They reported no significant differences in early clinical or radiographic outcomes among the groups. The respective complication rates were 3.6% for full-length stems, 5.9% for reduced distal profile stems, and 9.1% for short-length stems. Although numerically higher in the short stem group, this difference did not reach statistical significance.

The advantage of the shortened stem used in this study is that it has the same design and zonal fixation as a standard stem. All the short stems featured a metaphyseal-diaphyseal fixation, combining the advantage of sharing the same design as standard stems while eliminating the drawbacks of increased length. Importantly, no short stems with proximal (calcar-based) anchorage were included, as these typically have a particular design associated with distinct and occasionally significant complications. One of the specific challenges associated with the DAA without a traction table is femoral exposure, which often necessitates the use of specialized instruments and, occasionally, a muscular release. While there is a risk of GT fracture during this process, suboptimal femoral exposure may increase the risk of GT fragility during stem implantation. A shortened stem reduces the constraints applied to the femur during the stem preparation during DAA without a traction table. Nevertheless, the occurrence of such fractures typically does not impact postoperative outcomes. Full weight-bearing is generally allowed, and surgical revision is rarely required in most cases.

This study revealed a slightly higher prevalence of varus alignment in the shortened stem group than in the standard stems, with a difference of 0.1°. However, this difference is not clinically significant. This discrepancy in alignment patterns raises questions regarding the factors influencing alignment outcomes with different stem designs, including surgical technique, implant characteristics, and patient-specific factors. The association between femoral stem alignment and postoperative complications is a hot topic in THA research. In this study, the femoral complications predominantly occurred with femoral stem alignment in a neutral position. Factors beyond alignment, such as implant design, bone quality, and surgical technique, may play significant roles in determining the occurrence of complications.

The design of shortened stems allows for adaptation to anatomy and preservation of femoral offset; they reported that varus positioning could sometimes be related to femoral morphology, particularly coxa vara deformity, and was not necessarily a technical failure [26]. Surgeons can better respect the patient’s native anatomy, positioning the femoral stem in the varus. Since the stems are shorter, diaphyseal fixation is reduced as the stem is less aligned with the axis. Nishioka et al. conducted a retrospective study of 441 THA performed with DAA using the same cementless tapered-wedge shortened stem to evaluate whether varus malalignment contributes to the risk of early periprosthetic fractures. They found that 19.6% of the stems were placed in varus alignment, and three periprosthetic fractures occurred in neutrally aligned stems. Additionally, only one case of aseptic loosening was associated with malalignment. These findings suggest that while varus positioning may theoretically be a factor, forceful intraoperative realignment of a shortened femoral stem with good initial fixation could unnecessarily increase the risk of intraoperative fractures without providing significant clinical benefit [27]. Batailler et al. [28] analyzed these two stems using finite element analysis, finding no force on the distal part of the standard stem and, therefore, no significant impact on stresses. The metaphyseal design is the same for both stems, thus maintaining similar proximal fixation. In addition, Burchard et al. [29] performed a comparative finite element analysis and demonstrated that the degree of stress shielding varies significantly depending on both stem geometry and material stiffness. Their results showed that shorter stems with metaphyseal fixation and lower stiffness generated less proximal stress shielding compared to longer, stiffer diaphyseal-engaging stems. Although our study did not include radiographic evaluation of bone remodeling, the design of the shortened stem used, which eliminates the distal portion while preserving metaphyseal fixation, may theoretically help reduce proximal stress shielding. This potential advantage, particularly relevant in younger patients or those with good bone quality, warrants further radiographic investigation in long-term follow-up studies.

Nonetheless, the long-term consequences of varus positioning deserve attention. Masuda et al. [30] recently demonstrated, using a 3D-templating software to accurately measure alignment, that varus positioning of short tapered-wedge cementless stems was associated with continuous bone mineral density loss over a five-year follow-up, potentially affecting implant longevity. Similarly, Maeda et al. [31] showed that varus malalignment significantly impacted periprosthetic bone density in the Avenir Complete stem, especially in Gruen zones 1 and 7. Iwase et al. [32], studying cemented stems, reported distal femoral cortical hypertrophy in varus-aligned cases, suggesting altered load transfer and a possible risk of thigh pain. While these effects were not observed at one year in our series, they underscore the need for cautious alignment and long-term monitoring of varus-positioned stems.

Several studies indicate that varus alignment of the shortened stem is associated with poor clinical outcomes, an increased risk of complications [33], and more pain [34, 35]. Additionally, some authors have found an increased risk of periprosthetic fracture with a valgus alignment [36, 37]. Griffiths et al. [37] demonstrated that a varus angle greater than 5 degrees is a risk factor for periprosthetic fracture. The study included standard and shortened stems, but the femoral stem design did not demonstrate a significant association with fracture risk. This extreme value is rarely observed in our series of shortened stems. Nevertheless, not all designs of short or shortened stems are equivalent. It is crucial to identify the used stem well and not to generalize the conclusions.

Insufficient preparation of the lateral edge of the femoral diaphysis or the persistence of the lateral upper cortical bone of the femoral neck can lead to varus displacement, especially in cases of coxa vara deformity. With the DAA, femoral preparation is sometimes more challenging, and the malalignment risk is slightly higher than with the posterior approach [18]. For these more complex cases, optimal instruments for femoral preparation are necessary.

This study had certain limitations. Firstly, regarding the study design, it is a retrospective single-center study. The implants used may not necessarily be generalizable to all shortened stems. Additionally, this is a radiological analysis, and femoral rotation can impact the measure of stem alignment. Nevertheless, the CT scan is an irradiating exam, which is not recommended systematically. Complication assessment was based on postoperative radiographs, which made blinding of the assessors impossible due to the visible difference in stem length. However, the complications recorded, such as fractures or subsidence, were clearly defined and objectively identifiable, reducing the risk of interpretation bias. Unlike clinical scores, which may be influenced by subjective judgment, these radiographic findings represent robust and reproducible endpoints. Another limitation is that although matching was performed based on age, sex, and BMI, other potentially influential factors such as bone mineral density (e.g., T-score), preoperative functional status, or stem size were not included in the matching criteria due to inconsistent availability in retrospective records. These parameters could have influenced femoral complication rates and should be considered in future prospective studies. Then, the follow-up was short. The stem survival can’t be analyzed in this study and will necessitate further assessments in the future.

## Conclusion

Shortened stems in DAA THA were associated with a lower rate of femoral complications, particularly fewer GT fractures. Although shortened stems were more often positioned in varus, this did not impact short-term complication rates.

## Data Availability

The datasets used and/or analysed during the current study are available from the corresponding author on reasonable request.

## Abbreviations

BMI: Body Mass Index
DAA: Direct Anterior Approach
GT: Great Trochanter
THA: Total Hip Arthroplasty
